# Plasma Level of IL-4 Differs in Patients Infected with Different Modern Lineages of *M. tuberculosis*

**DOI:** 10.1155/2012/518564

**Published:** 2012-09-25

**Authors:** Adane Mihret, Yonas Bekele, Andre G. Loxton, Abraham Aseffa, Rawleigh Howe, Gerhard Walzl

**Affiliations:** ^1^Armauer Hansen Research Institute, P.O. Box 1005, Addis Ababa, Ethiopia; ^2^Department of Microbiology, Immunology and Parasitology, School of Medicine, College of Health Sciences, Addis Ababa University, P.O. Box 9086, Addis Ababa, Ethiopia; ^3^Division of Molecular Biology and Human Genetics, DST/NRF Centre of Excellence for Biomedical Tuberculosis Research, MRC Centre for Molecular and Cellular Biology, Faculty of Medicine and Health Sciences, Stellenbosch University, P.O. Box 19063, Francie van Zijl Drive, Tygerberg 7505, South Africa

## Abstract

Epidemiological evidence from tuberculosis outbreaks revealed that some genotypes of *M. tuberculosis* are more transmissible and capable of causing disease than others. We analysed the plasma cytokine levels of pulmonary tuberculosis patients infected with different strains of *M. tuberculosis* to test the hypothesis that immune responses would be linked to the bacterial genotype. Spoligotyping was carried out for genotyping, and we used Luminex technology to measure 17 cytokines (EGF, fractalkine, GM-CSF, IFN-**γ**, IL-1, IL-10, IL-12, IL-17, IL-4, IL-7, IL-9, IP-10, MCP-1, MCP-3, MIP-1**β**, TNF, and VEGF) from plasma samples of tuberculosis patients. The levels of IL-12 (p40), IL-4, IL-7, and MIP-1beta were higher in patients infected with lineage 3, however, it was only IL-4 which showed statistically significant difference (*P* < 0.05) between lineage 3 and lineage 4. We further grouped the lineages into families (CAS, H and T families), and we found that the plasma level of IL-4 was significantly higher in patients infected with the CAS family (*P* < 0.05) in comparison with T and H families. However, there was no difference between T and H families. Therefore, the higher level of IL-4 in lineage 3 families might indicate that possible differences in the response elicited from host depend on strain lineages in the studied population.

## 1. Introduction

Tuberculosis remains a significant public health problem, and it is estimated that one-third of the world's population is infected with *Mycobacterium tuberculosis*, although the majority will never develop active disease [1]. The factors that lead to the considerable variability in the outcome of *M. tuberculosis *infection are complex and incompletely understood. Host genetics and environmental factors such as prior exposure to nonpathogenic mycobacteria, HIV infection, advanced age, malnutrition, alcohol abuse, diabetes, and use of corticosteroids have been associated with tuberculosis disease [2]. Moreover, as tuberculosis disease results from the interactions between host and bacteria, there is growing evidence that the genetic diversity of *Mycobacterium tuberculosis *may have important clinical consequences [3, 4]. 

The global population structure of *M. tuberculosis *is defined by six phylogeographical lineages: Indo-Oceanic lineage, East Asian lineage, East African Indian lineage, Euro-American lineage, West African lineage I and West African lineage II [5]. The Indo-Oceanic lineage (lineage I), West African lineage I (lineage 5), and West African lineage II (lineage 6) are belonging to ancient lineages whereas the East Asian lineage (lineage 2), East African-Indian lineage (lineage 3) and Euro-American lineage (lineage 4) are belonging to the modern lineage [6]. Epidemiological evidence from tuberculosis outbreaks suggested that some genotypes of *M. tuberculosis *are more transmissible and more capable of causing disease than others and that some genotypes of *M. tuberculosis *may be associated with tuberculosis affecting different organs [6, 7]. A number of studies have described the “Beijing family” to be hypervirulent with a reduced immune response leading to higher bacillary load and enhanced dissemination with rapid progression to severe disease in humans and experimental animals [8–11].

A study in Gambia showed that progression to clinical disease was significantly less in individuals exposed to the ancient lineage compared to the modern lineage [12], and another study in Madagascar reported significantly lower interferon-*γ*  production by peripheral blood T cells in individuals infected with the modern lineage [13]. Moreover, a recent study by Portevin et al. also showed that measurement of cytokines from culture supernatants harvested 24 hours after infection of human peripheral blood monocyte-derived macrophages revealed clear differences in the level of pro-inflammatory cytokines produced by a single donor in response to different strains [14]. 

In the present study, we used plasma samples from newly diagnosed pulmonary tuberculosis patients to test the hypothesis that immune response would be linked to the infecting genotype by measuring the plasma levels of different cytokines and chemokines.

## 2. Materials and Methods

### 2.1. Patient Recruitment and *M. tuberculosis* DNA Separation

A total of 75 HIV-negative microbiologically confirmed new smear-positive pulmonary TB patients attending Arada, T/Haimanot, Kirkos, and W-23 health centres, Addis Ababa and who were infected with one of the three major families, T, H, and CAS, were selected from the total of 192 patients. The diagnosis of TB in the health centers was based on the national guidelines of at least two positive smears for acid-fast bacilli (AFB) in three specimens collected from each patient as spot-morning-spot. All sputum samples from TB cases were cultured for mycobacteria. The presence of HIV infection was ruled out using rapid tests (Stat pack, KHP, and Unigold as a tie breaker) as per the national guideline. The modified Petroff's method was used to digest and decontaminate the sputum specimens. An aliquot of 100 *μ*L of the sample was then inoculated onto two Löwenstein-Jensen (LJ) slants. Bacterial growth was read every week up to 8 weeks. Cultures with no growth after the 8th week were considered negative. *M. tuberculosis *isolates were identified using PCR-based genotyping with previously described methods for RD9 deletions [15]. Mycobacterial genomic DNA was extracted by heating the isolates at 80°C for 60 min and was stored at −20°C until needed for spoligotyping. The study obtained an institutional ethical clearance from AHRI/ALERT ethics committee (Reference no. p015/10) and a national ethical clearance from National Research Ethics Review Committee (NRERC) (Reference 3.10/17/10). We sought a written informed consent from all participants.

### 2.2. Spoligotyping

Spoligotyping was carried out using the commercially available kit from Ocimum Biosolutions, India, according to the manufacturer's instructions. Briefly, the direct-repeat (DR) region was amplified with primers DRa (biotinylated at the 5′ end) and DRb, and the amplified DNA was hybridized to inter-DR spacer oligonucleotides covalently bound to a membrane. DNA from *Mycobacterium bovis *BCG and *M. tuberculosis *H37Rv was used as a positive control, whereas autoclaved ultrapure water was used as a negative control. The amplified DNA was subsequently hybridized to a set of 43 oligonucleotide probes by reverse line blotting. The presence of spacers was visualized on film as black squares after incubation with streptavidinperoxidase and detected with the enhanced chemoluminescence system detection liquid (Amersham, Little Chalfont, UK). 

### 2.3. Cytokine Measurement

We used a 17plex kit (epidermal growth factor (EGF), FRACTALKINE, granulocyte macrophage colony-stimulating factor (GM CSF), IFN-*γ*, IL-1, IL 10, IL-12, IL-17, IL-4, IL-7, IL-9, IFN-*γ*-inducible protein (IP-10/CXCL-10), Macrophage chemoattractant protein 1 (MCP-1/CXCL), MCP-3, monocyte inflammatory protein 1 beta (MIP-1*β*), TNF, and VEGF) from Millipore, Germany, and multicytokine analysis was done using Luminex (Millipore, Germany) technology. The principle of the technique is based on developing color-coded microspheres by combining different ratios of two dyes, and this combination can give upto 100 different combinations, which enables to measure 100 analytes. The technology combines flow cytometry and ELISA together where the capture antibody is conjugated with beads or microspheres, whereas the secondary antibody is conjugated with fluorochrome which quantifies the antigen-antibody reaction by measuring the relative fluorescence intensity. The assays were performed according to the supplier instruction. Briefly, following prewetting of plates, 50 *μ*L precombined beads of all the 17 individual cytokines or chemokines were added and washed twice. Plasma samples (25 *μ*L) were diluted 1 : 1 with the kit serum matrix and added to the plate. The plate was shaken for 30 sec at 1000 RPM and then incubated for 1 hr on plate shaker at 300 RPM at room temperature. Plates were washed twice, and 25 *μ*L of detection antibody was added per well and incubated for one hour on a plate shaker. Fifty microliters of a streptavidin-PE conjugate was added per well and incubated for 30 min at room temperature. Finally, plate was washed three times, and 150 microliter of sheath fluid was added to each well, and then the plate was read by Luminex machine, and data was analysed by Luminex 100 IS software version 2.3.182.

### 2.4. Statistical Analysis

The data were analyzed using Graphpad prism software, version 4.0. Nonparametric Mann-Whitney  *U*  tests were performed to test for the significance of the observed differences in each parameter in TB and other groups. A *P* value less than 0.05 was considered statistically significant.

## 3. Result

### 3.1. Genetic Diversity and Family Assignment

**Figure 1:**
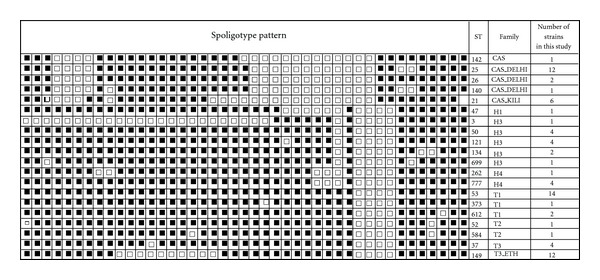
Spoligotype pattern of *M. tuberculosis* strains. The black and white boxes indicate the presence and absence, respectively, of the specific spacer at position 1 to 43 in the DR locus. CAS = Central Asian; T = ill-defined family; H = Haarlem.

A total of 75 TB patients infected with *M. tuberculosis* belonging to lineages 3 and lineage 4 were selected randomly from 192 patients. The lineage 3 comprises 22 CAS families, and 4 comprises 35 T and 18 H families. Among the 75 patients, 26 (34.6%) were females. The mean age was 28.7 years (range 18–64) and 31.7 years (range 18–59) for females. The representative spoligopattern is indicated in Figure 1. 

### 3.2. Plasma Cytokine and Chemokine Levels of Patients Infected with Different *M. tuberculosis* Lineages

**Figure 2:**
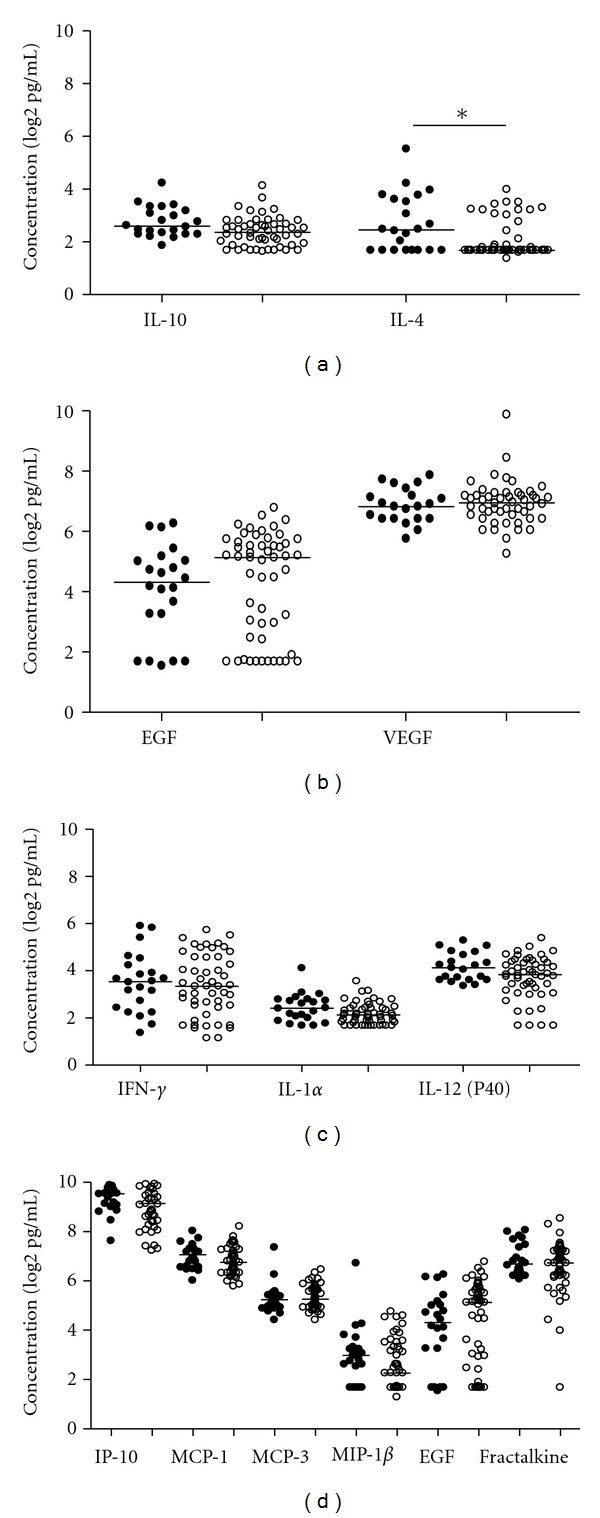
Plasma cytokine and chemokine levels in TB cases infected with different lineages. Plasma samples from TB cases infected with lineage 3 (*n* = 22) and lineage 4 (*n* = 43) were assessed by multiplex cytokine analysis. (a) Anti-inflammatory cytokines (IL-10 and IL-4), (b) growth factors (EGF and VEGF), (c) pro-inflammatory cytokines (IFN-*γ*, IL 12 (p40), and TNF) and (d) chemokines (IP-10, MCP-1, MCP-3, MIP-1*β* and fractalkine). Horizontal line indicates median levels of TB cases infected with lineage 3 (filled circles) and lineage 4 (open circles). data were analysed using nonparametric Mann-Whitney test with *P* values indicating significant differences after data were trimmed and transformed to Log 2 values. *P < 0.05.

Plasma samples from 75 TB patients infected with strains belonging to lineage 3 (CAS) (*n* = 22) and lineage 4 (H and T) (*n* = 43) were analysed, and we compared the plasma cytokine and chemokine levels between the two lineages. The levels of IL-12 (p40), IL-4, EGF, IP 10 and MIP-1*β* were higher in patients infected with lineage 3; however, it was only IL-4 which was significantly different between the two lineages (*P* < 0.05) (Figure 2).

### 3.3. Plasma Cytokine and Chemokine Level of Patients Infected with Different *M. tuberculosis* Families

**Figure 3:**
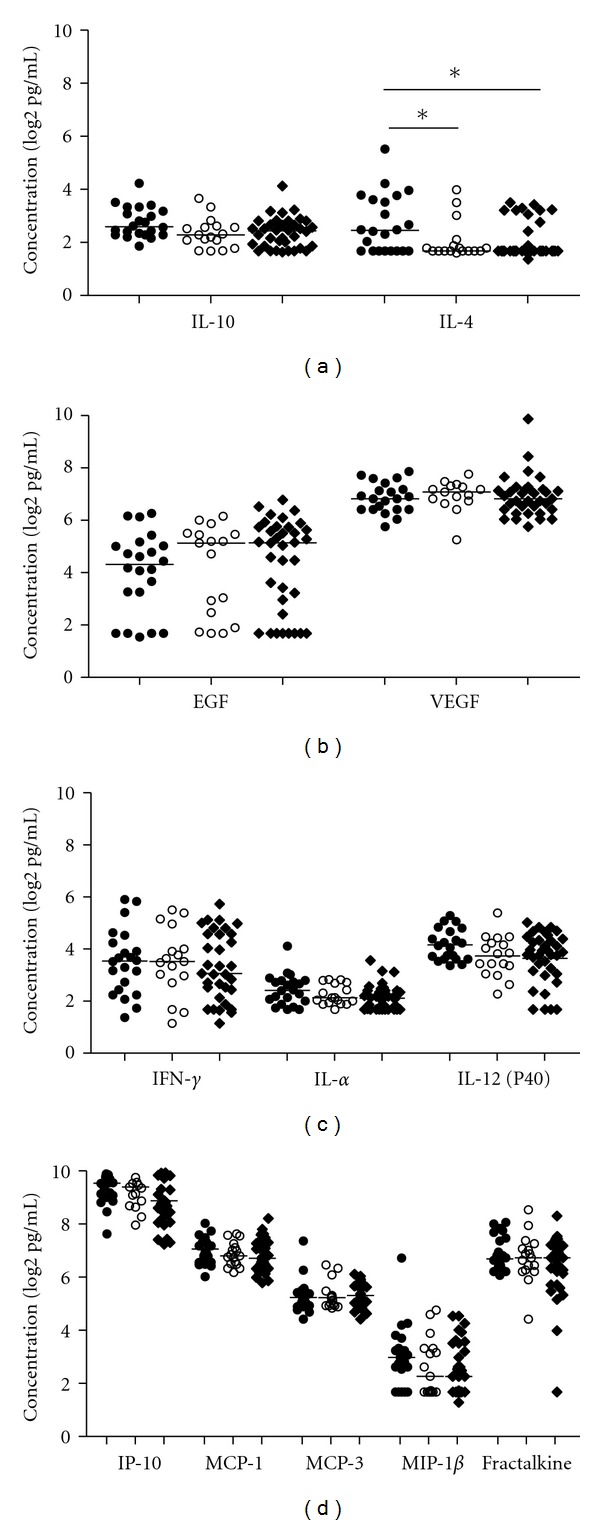
Plasma cytokine and chemokine levels in TB cases infected with different strains. Plasma samples from TB cases infected with CAS (*n* = 22), H (*n* = 18), and T families (*n* = 35) were assessed by multiplex cytokine analysis. (a) Anti-inflammatory cytokines (IL-10 and IL-4), (b) growth factors (EGF and VEGF), (c) Proinflammatory cytokines (IFN-*γ*, IL-12 (p40), and TNF) and (d) chemokines (IP-10, MCP-1, MCP-3, MIP-1*β*, and fractalkine). Horizontal line indicates median levels of TB cases infected with the CAS family (filled circles), H family (open circles) and T family (filled diamonds). Data were analysed using nonparameteric Mann-Whitney test with *P* values indicating significant differences after data were trimmed and transformed to Log2 values. *P < 0.05.

We further grouped the lineages into different *M. tuberculosis* families: 22 CAS, 35 T, and 18 H families, and we found that only the level of IL 4 was significantly higher in plasma samples infected with CAS family (*P* < 0.05) in comparison with H and T families. However, there was no difference between T and H families (Figure 3).

**Figure 4:**
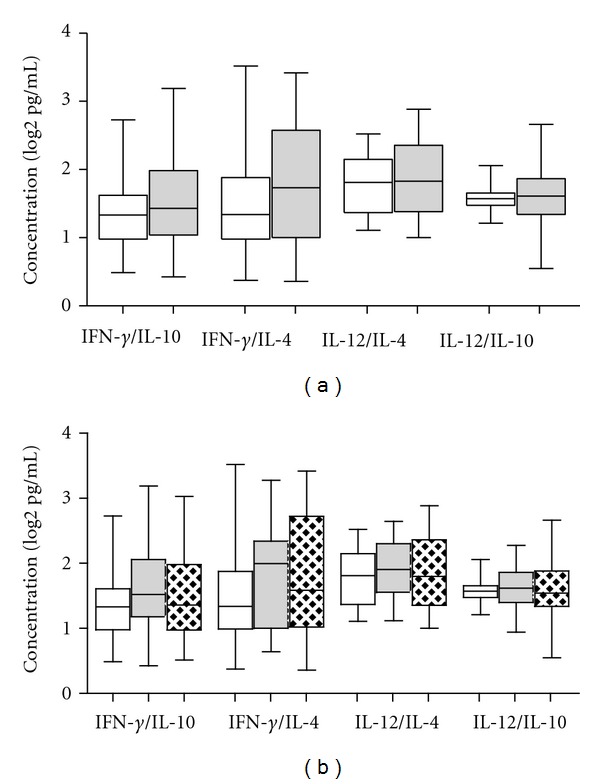
Th1/Th2 ratios of cytokines of TB cases infected with different strains. Box plots are shown with the horizontal line indicating median levels of TB cases and the lower and upper edges of each box indicate the 25th and 75th percentiles, respectively. (a) Ratio of Th1/Th2 cytokines (IFN-*γ*/IL-4, IFN-*γ*/IL-10, IL-12/IL-4, and IL-12/IL-10) of TB patients infected with lineage 3 (white bars), and lineage 4 (grey bars); (b) Ratio of Th1/Th2 cytokines (IFN-*γ*/IL-4, IFN-*γ*/IL-10, IL-12/IL-4, and IL-12/IL-10) of TB patients infected with CAS family (white bars) H family (grey bars), and T family (crossed bars). Data were analysed using nonparametric Mann-Whitney test with *P*-values indicating significant differences after data were trimmed and transformed to Log 2 values. *P < 0.05.

We also analysed the ratio of the main Th1 (IFN-*γ* and IL-12 (p40)) and Th2 (IL-4 and IL-10) hallmark cytokines, however, none of the ratios of IFN-*γ*/IL-4, IFN-*γ*/IL-10, IL-12 (p40)/IL-4, and IL-12 (p40)/IL-10 were significantly different between patients infected with the different lineages or families (Figure 4).

## 4. Discussion

Understanding the effect of the genotype of the infecting organism in the pathogenesis of tuberculosis is becoming an essential question in tuberculosis research. It is well known that T-cell responses play a fundamental role during *M. tuberculosis* infection where a strong T-cell response leads to granuloma formation and maintenance, whereas a defective T-cell response favors progression. In the present study, we analysed the plasma level of different cytokines and chemokines in TB cases who are diseased with different *M. tuberculosis *genotypes common in Addis Ababa, Ethiopia. 

Strains belonging to the modern lineage were the only *M. tuberculosis* isolates circulating in the study community, and we compared the plasma level of cytokines and chemokines of people infected with the different lineages and families within the modern lineage. Previous studies using laboratory and clinical strains have shown differences in immune response amongst *M. tuberculosis* isolates. For example, strain NH878 has been associated with a low inflammatory immune response and increased virulence in macrophages and animal models compared to H37Rv, H37Ra, Erdman, and CDC1551 [16–19]. A recent study also showed a wide variation in the immune response after measurement of cytokines from infected human peripheral blood monocyte-derived macrophages where modern lineages induced lower inflammatory responses in comparison with ancient lineage. This lower immune response might promote more rapid disease progression and increase transmission in case of modern lineages [14]. 

In our study, we compared the plasma level of 17 cytokines, which include proinflammatory cytokines, anti-inflammatory cytokines, angiogenic factors, and chemokines, in tuberculosis patients infected with different *M. tuberculosis* strains of the modern lineage. The plasma level of IL-9, IL-17, IL-7, TNF, and GM-CSF was present in very low concentrations in all patients infected with different strains. Although detectable, no significant difference in levels of EGF, fractalkine, IFN-*γ*, IL-1, IL-10, IL-12 (p40), IL-7, IP-10, MCP-1, MCP-3, MIP-1*β*, and VEGF was found between patients infected with different lineages and families. We found that only the plasma level of IL-4 was significantly higher in patients infected with lineage 3 (*P* < 0.05) as compared to lineage 4. We further grouped the lineages into families, and similarly, it was IL-4 which showed statistical difference between the different families where patients infected with CAS family had a higher plasma level of IL-4 (*P* < 0.05) as compared to patients infected with H and T families but there was no difference between H and T families. 

Previous studies reported lower inflammatory response of modern strains including Beijing and other strains [10, 14, 20] where low inflammatory response was linked to increased virulence [8, 21]. One explanation could be that a reduction in innate immune recognition will result in a delay in engagement of the adaptive response, providing the pathogen with a significant advantage during the early stage of infection. Another study in Madagascar also showed that tuberculosis patients and their contacts who are infected with modern *M. tuberculosis *strains, like Beijing and Central Asian (CAS) strains, tended to induce lower IFN-*γ* responses than ancient strains, like East African-Indian (EAI) strains. 

Although we did not compare the two broad lineages of *M. tuberculosis*, ancient and modern lineages, we clearly saw a marked difference in the plasma level of IL-4 within families of the modern lineage. IL-4 is an anti inflammatory cytokine, and there are hypotheses that maintenance of a prolonged Th1 response against *M. tuberculosis *requires not only elevation of IFN-*γ*, but also downmodulation of the Th2 response, specifically IL-4. IL-4 suppresses macrophage derived production of IL-12, thereby inhibiting differentiation of Th1 cells, and inhibits cell mediated immune reactions by antagonizing the macrophage-activating effect of IFN-*γ*. Therefore, the higher level of IL-4 in lineage 3 families might indicate that possible differences in the response elicited from host depend on strain lineages in studied population. This present study is too small and was not designed to allow the detection of clinical differences between infections with different strains, including extent of disease, presence of cavitation, and treatment response but should be investigated in future studies.
